# Malignant external auditory canal and temporal bone tumours: Clinical outcomes in Cape Town, South Africa

**DOI:** 10.4102/jcmsa.v4i1.327

**Published:** 2026-06-24

**Authors:** Hervin N. Heeroo, Tashneem Harris, Matthew White, Sameera Dalvie

**Affiliations:** 1 Division of Otorhinolaryngology, Faculty of Health Sciences, University of Cape Town, Cape Town, South Africa; 2 Division of Radiation Oncology, Faculty of Health Sciences, University of Cape Town, Cape Town, South Africa

**Keywords:** external auditory canal, temporal bone cancer, tumour, carcinoma, malignancy, squamous cell carcinoma, temporal bone resection, survival, outcome

## Abstract

**Background:**

Temporal bone cancers are associated with high morbidity and poor prognosis. However, no established treatment guidelines currently exist. Information on temporal bone carcinomas in sub-Saharan Africa is almost non-existent in the literature. The aim of the study was to look at the outcomes of temporal bone malignancies in a developing-world setting.

**Methods:**

A retrospective chart review was conducted at a tertiary-care hospital in Cape Town, South Africa, of all patients with newly diagnosed temporal bone malignancies from 2008 to 2023. Distant metastases to the temporal bone were excluded.

**Results:**

In total, 20 patients were identified, with a mean age of 60.7 years. From the study sample, 40% of the patients were male, and 60% were female. The most common histological diagnosis was squamous cell carcinoma (70%), followed by adenoid cystic carcinoma (15%). Seventy per cent of patients were treated with curative intent; 65% with primary surgery and 5% with primary radiotherapy, respectively. The remaining patients were offered palliative radiotherapy (25%) and best supportive care (5%). Disease recurrence occurred in 25% of patients, with the mean time to recurrence being 5.6 months. The mean overall survival for the cohort was 1.52 ± 1.23 years, while the mean disease-specific survival was 1.68 ± 1.67 years.

**Conclusion:**

Early diagnosis of temporal bone cancers is fundamental, and aggressive resection is essential, if indicated, to obtain negative margins and minimise recurrence.

**Contribution:**

This study provides valuable insights into temporal cancers in sub-Saharan Africa and paves the way for larger studies on the continent.

## Introduction

Temporal bone carcinomas (TBCs) are extremely rare, constituting less than 0.2% of all head and neck cancers, with a worldwide annual incidence of 1–6 cases per million people.^1^ They are locally aggressive with an associated high morbidity and overall poor prognosis in advanced cases.^2^ Squamous cell carcinoma (SCC) is the most common subtype, accounting for 60% – 80% of cases,^3^ followed by basal cell carcinoma and adenoid cystic carcinoma.^4^ Temporal bone tumours can be primary or secondary. Primary tumours originate from the external auditory canal, middle ear, mastoid and petrous apex. Secondary involvement of the temporal bone by adjacent tumours of the parotid and surrounding tissues occurs more frequently.^5^ Distant metastases to the temporal bone from the breast, lung and prostate have also been described in the literature.^6^ Patients are commonly diagnosed in their sixties and typically present with non-specific symptoms of otorrhoea, otalgia and hearing loss, with or without facial nerve palsy.^7^ In the primary care setting, persistence of symptoms with aural toilet and antibiotics, and a thorough otomicroscopic examination should prompt suspicion of an ear malignancy and mandate a biopsy.^8^ Diagnosis involves biopsy, and radiological imaging is necessary for staging.^2^ Treatment modalities include surgery, radiotherapy, chemotherapy or a combination of these options.^9^

The Modified Pittsburgh Staging System is the most commonly used staging system for primary temporal bone cancers, specifically for SCC.^10^ Primary temporal bone cancers are classified into four prognostic stages depending on the extent of local cancer spread from the external ear canal (T-Stage), the degree of cervical lymph node involvement (N-Stage) and the presence or absence of distant metastases (M-Stage). Stage I disease occurs when TBCs are limited to the external auditory canal (EAC) without bony erosion or evidence of soft tissue involvement. Stage II disease denotes TBCs limited to the EAC with partial-thickness bone erosion or limited soft tissue involvement (< 0.5 cm). Stage III disease involves full-thickness erosion of the osseous EAC with limited soft tissue involvement (< 0.5 cm), or tumour involvement of the middle ear and/or mastoid. Stage IV disease with no nodal involvement occurs when there is erosion of ‘the cochlea, petrous apex, medial wall of the middle ear, carotid canal, jugular foramen or dura, with extensive (> 0.5 cm) soft tissue involvement or evidence of facial paresis’.^11^ However, any lymph node involvement or distant metastasis immediately signifies Stage IV disease and indicates a poorer prognosis.^12^

No established treatment guidelines presently exist for TBCs, given the rarity of cases.^13^ The primary curative intent involves surgically removing the tumour with clear margins, with or without adjuvant therapy.^14^ Treatment is mainly guided by tumour stage and patient comorbidities.^15^ En bloc lateral temporal bone resection (LTBR) is typically indicated for early-stage temporal bone cancers. Subtotal and total temporal bone resection are reserved for very advanced disease and are much less commonly performed.^12^ Facial nerve sacrifice is indicated when tumour directly involves or encases the facial nerve, resulting in facial nerve weakness. Facial nerve grafting with either the sural nerve or the greater auricular nerve can be achieved when proximal and distal nerve stumps are intact.^16^ Parotidectomy is indicated if there is direct invasion of the parotid gland through the anterior ear canal or where parotid lymph node metastases are present.^17^ Reconstruction and obliteration of an open cavity is typically required using local tissue transfer, pedicled flap or free flap techniques.^8^ A concurrent ipsilateral neck dissection is indicated in the presence of clinically or radiologically positive lymph node metastases. However, the management of the clinically occult N0 neck is controversial.^18^ The roles of adjuvant and neoadjuvant therapies in the treatment of advanced disease remain unclear.^3^ Future research should focus on immune checkpoint inhibitors such as pembrolizumab and nivolumab for patients with surgically resectable advanced TBCs.^19^ Neoadjuvant immunotherapy may help decrease the bulk of disease pre-operatively and offer better chances at achieving negative resection margins.^19^ The pathological response could be used to prognosticate and perhaps identify those patients who require additional therapy.

The rarity of temporal bone tumours can be confirmed by the fact that there are only a few case series published on temporal bone SCC. Non-squamous TBC reports are even rarer, being limited to case reports. At the time of the writing of this article, there was only one report from Sub-Saharan Africa in the literature, of two cases of temporal bone SCC.^20^ Because of the dearth of publications on TBCs, it has been systematically difficult to formulate an evidence-based management protocol. Our study is therefore an endeavour to systematise the surgical and oncological outcomes of patients with TBCs in a developing-world setting.

## Research methods and design

### Patient selection

The study was conducted at Groote Schuur Hospital, a tertiary institution in Cape Town, South Africa, that serves a population of approximately 1.5 million people.^21^ After receiving ethics approval from the Groote Schuur Hospital and institutional approval from Groote Schuur Hospital, patients were identified by searching hospital records for all patients with newly diagnosed temporal bone malignancy from 2008 to 2023. Data could not be collected prior to 2008 due to incomplete paper-based records. Inclusion criteria consisted of all patients with newly diagnosed temporal bone malignancies on histology. Exclusion criteria were tumours originating from the periauricular skin without involvement of the temporal bone, and metastases to the temporal bone from distant sites. Fifty-eight patients were identified from the hospital electronic patient database. Chart records were searched, and after applying the exclusion criteria described above, 38 patients were excluded, leaving 20 patients for analysis. Patient selection was cross-verified by the second author.

### Data extraction

A retrospective chart review was performed. Patients were reviewed for age, sex, histological diagnosis, date of diagnosis, anatomic site involvement, margin status, facial nerve involvement, dural involvement, bone invasion, perineural invasion, lympho-vascular invasion, staging, treatment, smoking history, alcohol intake, immune status, history of prior radiation, date of tumour recurrence and date of death. The Modified Pittsburgh Staging System was used to stage patients. Treatment received consisted of surgery (primary, adjuvant and reconstructive), radiotherapy (primary and adjuvant), adjuvant chemotherapy or palliative care. Margin status was based on post-surgical pathology reports. Disease-free survival was determined from the date of tumour recurrence or the date of death. Disease-specific survival and overall survival were determined from the date of death. Data were collected on a Microsoft Excel spreadsheet and were stored on a password-protected computer.

### Multi-disciplinary team

At our institution, treatment plans are discussed at a multi-disciplinary tumour board once a histological diagnosis from a biopsy is obtained. Tumour resection is guided by the extent of the disease and intraoperative frozen section. Indications for adjuvant radiotherapy include advanced Stage 3 or 4 disease, margin positivity, positive nodal disease and extra-nodal extension.

### Surgical resection

At our unit, surgical resection of T1 temporal bone tumours involving the cartilaginous EAC is achieved through sleeve resection. Where the tumour extends into the bony EAC, LTBR is performed. T2 tumours are resected through LTBR. While T3 tumours may be amenable to LTBR, T4 tumours are not considered suitable for surgery. Neck dissection is performed in patients who have clinical or radiographic evidence of cervical lymph nodes. For SCC without nodal metastasis, neck dissection of levels 1b, 2–5 is considered. In patients with gross parotid involvement, parotidectomy is done to obtain negative margins, particularly with tumour extension into the anterior ear canal, or in case of lymphatic spread to the parotid gland. However, elective parotidectomy remains controversial. For basal cell carcinoma (BCC) without parotid invasion, parotidectomy is not indicated. If the tumour extends into or near the temporomandibular joint, a condylectomy or partial mandibulectomy is performed.

### Surgical reconstruction

In our hospital, the type of reconstruction is dependent on the extent of the defect. If the concha and tragus are resected but the pinna is spared, reconstruction is done using a temporalis muscle flap which is rotated over the defect and sutured to the sternocleidomastoid muscle. If a small defect is present, the temporalis muscle is covered with a split skin graft. For larger tumours where the tumour extends beyond the pinna into surrounding skin and soft tissue, a free flap is required.

### Statistical analysis

Descriptive statistics were used to analyse the dataset. The interquartile range was calculated for age. Kaplan–Meier survival analyses were performed, and 2-year survival estimates were extrapolated for outcomes of interest (overall survival, disease-specific survival and disease-free survival). A *p* value of less than 0.05 was considered statistically significant. Statistical analyses were performed using RStudio 2024.12.1.

### Ethical considerations

Ethical clearance to conduct this study was obtained from the University of Cape Town Human Research Ethics Committee (No. 674/2023).

## Results

A total of 20 patients were identified with a median age of 61.4 years at diagnosis (range = 31.5–89.5 years, interquartile range = 17.5 years). Our sample consisted of eight males (40%) and 12 females (60%) (Table 1). In all, 55% of patients were smokers (*n* = 11/20), and 25% consumed alcohol regularly (*n* = 5/20). Of note, all those who consumed alcohol were also smokers. All 20 cases were primary temporal bone cancers. The most common histological diagnosis was SCC (70%, *n* = 14/20), followed by adenoid cystic carcinoma (15%, *n* = 3/20). There was one case each of basosquamous carcinoma, BCC and acinic cell carcinoma. The most common site of involvement was the EAC (75%, *n* = 15/20), followed by the temporal bone (25%, *n* = 5/20). Facial nerve and dural involvement were each noted in 25% of cases (*n* = 5/20). Bone invasion was found in 55% of cases (*n* = 11/20). Perineural and lymphovascular invasion were found in 20% (*n* = 4/20) and 5% (*n* = 1/20) of cases, respectively. According to the Modified Pittsburgh Staging System, 15% (*n* = 3/20) of patients were Stage 1, 20% (*n* = 4/20) were Stage 2, 25% (*n* = 5/20) were Stage 3 and 40% (*n* = 8/20) were Stage 4. Sixty-five per cent (*n* = 13/20) and 5% (*n* = 1/20) of patients were offered primary surgery and radiotherapy, respectively. The remaining patients were offered palliative radiotherapy (25%, *n* = 5/20) and best supportive care (5%, *n* = 1/20), respectively.

**TABLE 1:** Demographic profile of participants, tumour characteristics and treatment offered.

Demographic profile	Total patients (*N* = 20)		
	Mean	*n*	%
**Age (years)**	61.4	-	-
**Sex**			
Male	-	8	40
Female	-	12	60
**Risk factors**			
Smoking	-	11	55
Alcohol	-	5	25
**Site of involvement**			
External auditory canal	-	15	75
Temporal bone	-	5	25
**Histological diagnosis**			
Squamous cell carcinoma	-	14	70
Adenoid cystic carcinoma	-	3	15
Basosquamous carcinoma	-	1	5
Basal cell carcinoma	-	1	5
Acinic cell carcinoma	-	1	5
**Pathological features**			
Facial nerve palsy	-	5	25
Dural involvement	-	5	25
Bone invasion	-	11	55
Perineural invasion	-	4	20
Lymphovascular invasion	-	1	5
**T-Stage**			
T1	-	3	15
T2	-	4	20
T3	-	5	25
T4	-	8	40
**N-Stage**	-		
N0	-	18	90
N1-3	-	2	10
**M-Stage**			
M0	-	19	95
M1	-	1	5
**Overall stage**			
Stage 1	-	3	15
Stage 2	-	4	20
Stage 3	-	5	25
Stage 4	-	8	40
**Treatment**			
Primary surgery	-	13	65
Primary radiotherapy	-	1	5
Palliative radiotherapy	-	5	25
Best supportive care	-	1	5

Of the 13 patients who had primary surgery, 69.2% (*n* = 9/13) had a LTBR, 23.1% (*n* = 3/13) had a sleeve resection, and 7.7% (*n* = 1/13) had a subtotal petrosectomy. Of the nine patients who had a LTBR, one patient was a T1, three patients were T2, and five patients were T3. Out of the three patients who had a sleeve resection, two were T1 and one was a T2. This T2 patient had circumferential soft tissue margins involvement by cancer and subsequently needed a LTBR, superficial parotidectomy and neck dissection. One T4 patient had a subtotal petrosectomy due to tumour invasion of the ear canal, mastoid cavity and infiltration along the sigmoid sinus and dura. Neck dissection was performed in 35% (*n* = 7/20) of patients who were all clinically N0. Out of these seven patients, five were squamous cell carcinomas, one was a basosquamous carcinoma, and one was a BCC. For SCC, levels 1, 2 and 3 were dissected in two patients, followed by levels 2 and 3 in two patients and levels 1, 2, 3, and 4 in one patient. Levels 2 and 3 were dissected for each of the basosquamous carcinoma and BCC, respectively. All the neck dissection specimens were negative for nodal metastasis. There were only two Stage 4 patients with clinical evidence of cervical lymphadenopathy. However, none of them was operable. Reconstruction of the surgical defects was performed in 11 patients. Reconstruction of the two patients with basosquamous carcinoma and BCC was performed using a radial forearm free flap, while reconstruction of the remaining nine patients was done with a temporalis muscle flap. All the patients who had facial nerve involvement were inoperable and received palliative radiotherapy, and hence, no patients required facial nerve repair or grafting. The details of the operations performed are presented in Table 2.

**TABLE 2:** Surgical procedures performed.

Surgical procedures	Patients	
	*n*	%
**Primary surgery**	13	100.0
Lateral temporal bone resection	9	69.2
Sleeve resection	3	23.1
Subtotal petrosectomy	1	7.7
**Lateral temporal bone resection**	9	-
T1	1	-
T2	3	-
T3	5	-
**Sleeve resection**	3	-
T1	2	-
T2	1	-
**Neck dissection (N0)**	7	-
Squamous cell carcinoma	5	-
Basosquamous carcinoma	1	-
Basal cell carcinoma	1	-
**Neck levels dissected (N0)**	7	-
2,3	4	-
1,2,3	2	-
1,2,3,4	1	-
**Reconstruction**	11	-
Temporalis muscle flap	9	-
Radial forearm free flap	2	-

Ten out of the 13 patients who were operated on had a parotidectomy, and all were superficial parotidectomies. All ten patients had direct involvement of the anterior ear canal, with tumour invasion of the parotid gland in three T3 cases (15%, *n* = 3/20). Of the latter three cases, there was only one case of an intra-parotid node which was positive for SCC (5%, *n* = 1/20). The details of tumour stage, parotid involvement and parotidectomy are presented in Table 3.

**TABLE 3:** Parotid involvement and parotidectomy.

Modified Pittsburgh Staging System	Overall stage	Parotid involvement (*n*)	Parotidectomy (*n*)
T1N0M0	Stage 1	0	1
T2N0M0	Stage 2	0	4
T3N0M0	Stage 3	3	4
T4N0M0	Stage 4	0	1

Resection margins were clear in 53.8% (*n* = 7/13) of patients and involved in 46.2% (*n* = 6/13) of patients. Eight per cent (*n* = 4/5) of the T3 tumours who had a LTBR had involved margins compared to only 20% (*n* = 1/5) of T1 and T2 tumours. Of the six patients with involved margins, there were three cases of squamous cell carcinomas, and one case each of adenoid cystic carcinoma, BCC and acinic cell carcinoma, as shown in Table 4. Disease recurrence occurred in 25% of patients (*n* = 5/20), with the mean time to recurrence being 5.6 months. All five patients were treated with surgery primarily and received adjuvant radiotherapy for involved margins. Of note, disease recurrence was noted in 80% of T3 tumours.

**TABLE 4:** Patients with involved margins.

Histology	Modified Pittsburgh Staging System	Surgery	Involved margins	Disease progression
Squamous cell carcinoma	T2N0M0	lateral temporal bone resection	Anterior and inferior margins involved	Right temporal lobe metastatic SCC
Adenoid cystic carcinoma	T3N0M0	lateral temporal bone resection	Residual tumour in the middle ear, involved soft tissue and bony resection margins	No
Basal cell carcinoma	T3N0M0	lateral temporal bone resection	Deep and posterior margins involved	No
Acinic cell carcinoma	T3N0M0	lateral temporal bone resection	One of the bone resection margins was involved by tumour, but due to the unorientated nature of the specimen, the exact margins could not be determined	Lung and brain metastases
Squamous cell carcinoma	T3N0M0	lateral temporal bone resection	Carcinoma extending to the inked excision margins	No
Squamous cell carcinoma	T4N0M0	subtotal petrosectomy	Superior, inferior, lateral and deep margins involved	Yes, with worsening ipsilateral earache and facial nerve palsy

SCC, squamous cell carcinoma.

Five out of those six patients with positive margins received adjuvant radiotherapy post-operatively. Disease progression was noted in a T2 SCC, a T3 acinic cell carcinoma and a T4 SCC despite adjuvant therapy. One of these five patients developed disease recurrence, with lung and brain metastases, and was offered best supportive care. The remaining patient (Stage 4) with positive margins, post-subtotal petrosectomy, received adjuvant chemoradiotherapy but due to disease persistence, went on to receive best supportive care. There was one patient with Stage 3 SCC, with clear margins post-LTBR, superficial parotidectomy and neck dissection levels 2 and 3, followed by adjuvant radiotherapy, who subsequently developed fixed lymph nodes in level 4 and 5, just below the radiotherapy field. This patient was given palliative radiotherapy. A total of 62.5% of patients with Stage 4 disease (*n* = 5/8) received palliative radiotherapy primarily. Out of these five patients, one patient received ‘holding’ chemotherapy while waiting for palliative radiotherapy. One patient with Stage 4 SCC received radiotherapy, primarily followed by palliative chemotherapy. Another patient with Stage 4 SCC was given best supportive care primarily. The details of adjuvant and/or palliative care given are presented in Table 5.

**TABLE 5:** Adjuvant and/or palliative treatment given.

Histology	Modified Pittsburgh Staging System	Margins	Adjuvant/Palliative treatment
Adenoid cystic carcinoma	T3N0M0, Stage 3	Involved	Adjuvant radiotherapy
Squamous cell carcinoma	T4N0M0, Stage 4	N/A	Palliative radiotherapy
Squamous cell carcinoma	T2N0M0, Stage 2	Involved	Adjuvant radiotherapy
Basal cell carcinoma	T3N0M0, Stage 3	Involved	Adjuvant radiotherapy
Squamous cell carcinoma	T4N0M0, Stage 4	N/A	Palliative radiotherapy
Squamous cell carcinoma	T4N0M0, Stage 4	N/A	Holding chemotherapy, Palliative radiotherapy
Acinic cell carcinoma	T3N0M0, Stage 3	Involved	Adjuvant radiotherapy, Best supportive care
Adenoid cystic carcinoma	T3N2aM1, Stage 4	N/A	Palliative radiotherapy
Squamous cell carcinoma	T4N0M0, Stage 4	Involved	Adjuvant chemoradiotherapy, Best supportive care
Squamous cell carcinoma	T3N0M0, Stage 3	Clear	Adjuvant radiotherapy, Palliative radiotherapy
Squamous cell carcinoma	T4N0M0, Stage 4	N/A	Best supportive care
Squamous cell carcinoma	T4N0M0, Stage 4	N/A	Palliative radiotherapy
Squamous cell carcinoma	T4N2bM0, Stage 4	N/A	Adjuvant radiotherapy, Palliative chemotherapy
Squamous cell carcinoma	T3N0M0, Stage 3	Involved	Adjuvant radiotherapy

N/A, not applicable.

**FIGURE 1:**
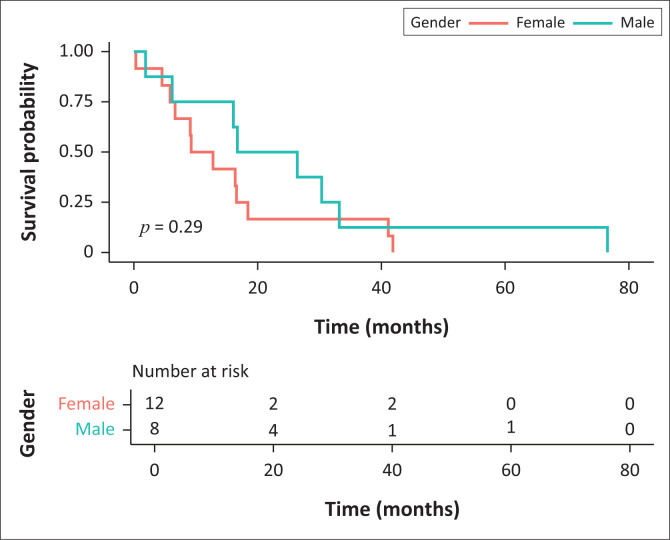
Kaplan–Meier curve of overall survival grouped by gender.

**FIGURE 2:**
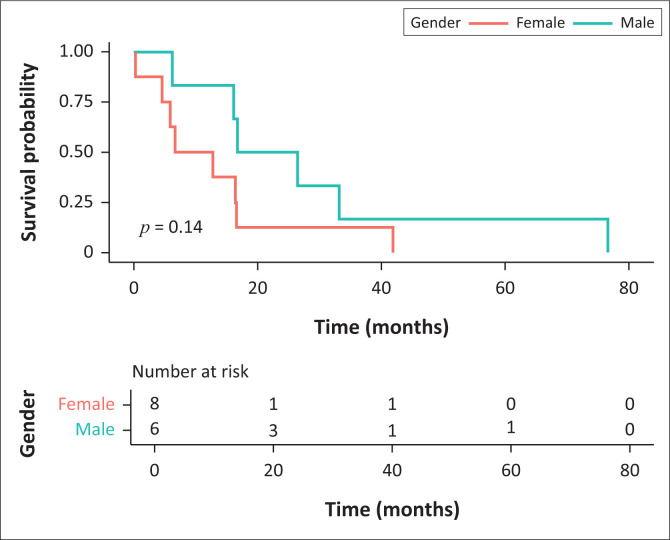
Kaplan–Meier curve of disease-specific survival grouped by gender.

**FIGURE 3:**
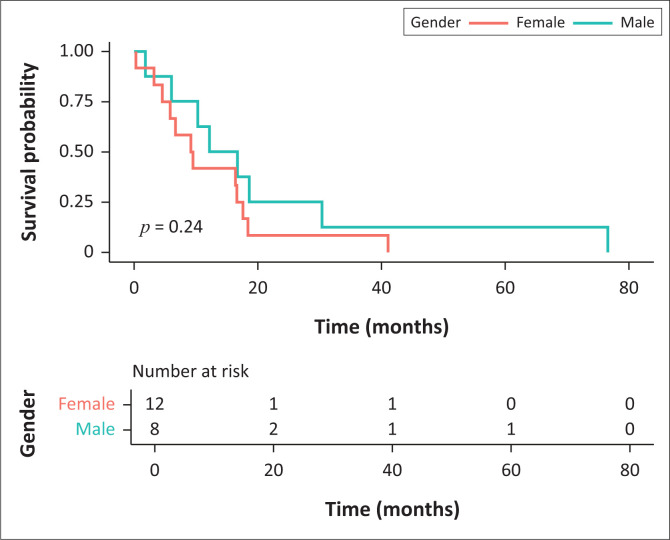
Kaplan–Meier curve of disease-free survival grouped by gender.

**FIGURE 4:**
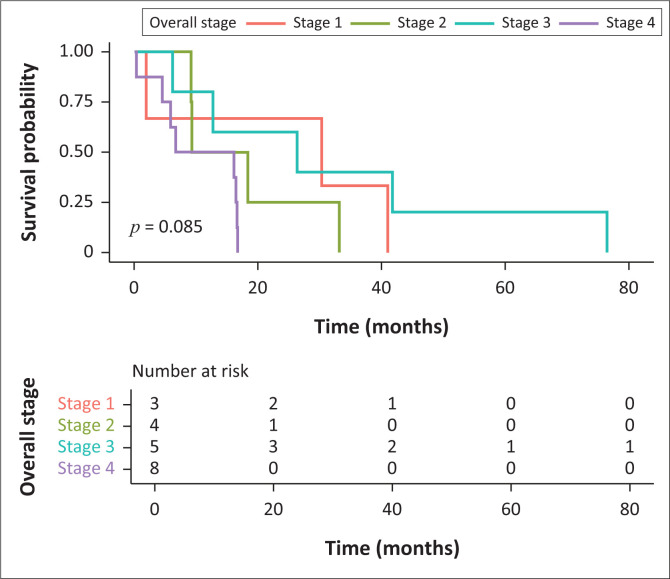
Kaplan–Meier curve of overall survival grouped by overall stage.

**FIGURE 5:**
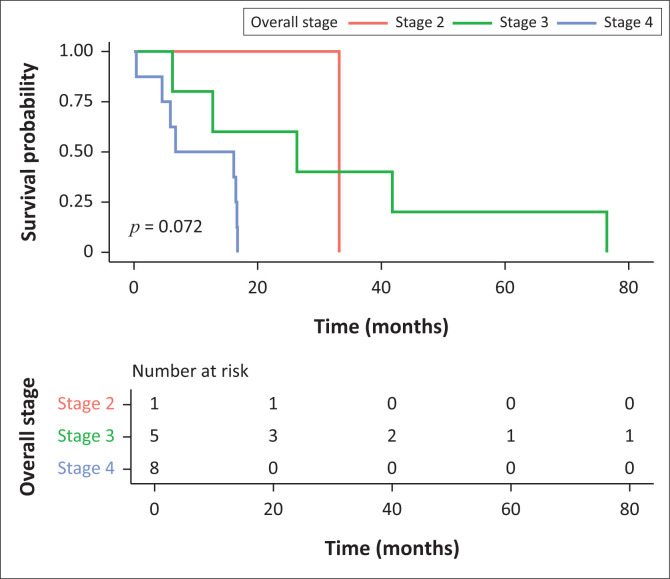
Kaplan–Meier curve of disease-specific survival grouped by overall stage.

**FIGURE 6:**
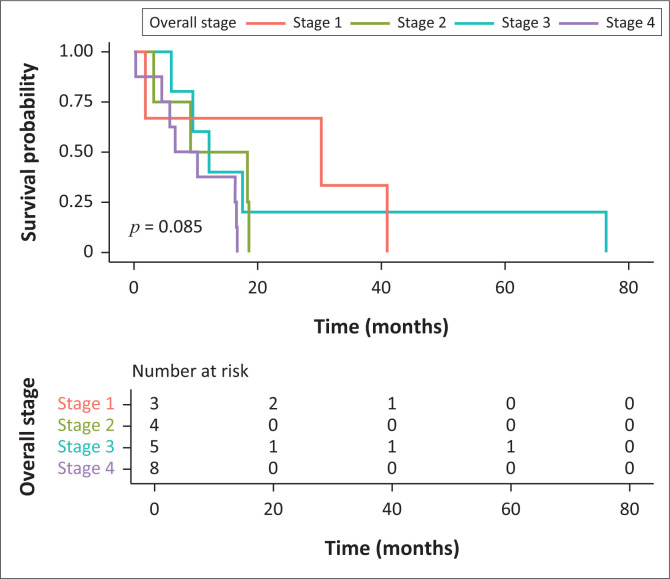
Kaplan–Meier curve of survival grouped by overall stage.

The mean overall survival for the cohort was 1.52 ± 1.23 years, while the mean disease-specific survival was 1.68 ± 1.67 years. The 2-year overall survival for T1, T2, T3 and T4 tumours were 66.7%, 25%, 60% and 0%, respectively. The 2-year disease-specific survival for T1–T2, T3 and T4 tumours were 100%, 50% and 0%, respectively. The 2-year disease-free survival for T1–T2, T3 and T4 tumours were 66.7%, 20% and 0%, respectively. Kaplan–Meier survival and log-rank analyses were performed for sex and stage of disease. For sex, statistical significance was not reached for overall survival (*p* = 0.29) (Figure 1), disease-specific survival (*p* = 0.14) (Figure 2) and disease-free survival (*p* = 0.24) (Figure 3). Similarly, for disease stage, statistical significance was not reached for overall survival (*p* = 0.085) (Figure 4), disease-specific survival (*p* = 0.072) (Figure 5) and disease-free survival (*p* = 0.24) (Figure 6).

## Discussion

Our study confirms the rarity of temporal bone cancers, as despite being a high-volume head and neck oncology unit, we were only able to identify 20 patients over a 16-year period. The median age at diagnosis was 61.4 years. This is in keeping with other studies where an older age at presentation is often seen.^5,9,21,22^ Our sample consisted of more females than males. However, in other studies, the majority of patients were found to be male.^5,23^ We found about half of our patients to be smokers and a quarter to be alcohol consumers. It is to be noted that, unlike other head and neck cancers, smoking and alcohol intake are not strongly linked with an increase in the risk of primary temporal bone SCC.^8^

In our study, the most common histologic type was SCC, followed by adenoid cystic carcinoma. Basosquamous carcinoma, BCC and acinic cell carcinoma were the least reported subtypes. This parallels existing literature reflecting that SCC is the commonest subtype, followed by BCC and adenoid cystic carcinoma.^8,9,12,24^ We found that the most common site of involvement was the EAC, followed by the temporal bone, similar to other studies.^1,9,25,26^ Facial nerve palsy, dural involvement and bone invasion rates in our study were higher than those of a study by Schachtel et al.,^27^ while perineural and lymphovascular invasion rates were lower. However, they included primary temporal bone SCC as well as cutaneous SCC originating from adjacent sites, which could explain the difference in findings.^27^ Most of our patients presented in advanced stages of the disease, with Stage 4 (Modified Pittsburgh Staging System) being the commonest stage of presentation, a finding that is repeated across multiple studies.^1,15,23,28^ One could believe that more advanced cases are seen in South Africa due to local resource limitations and the constraints of our referral system. However, as demonstrated by Piras et al., who performed their study at a quaternary referral centre in Italy, a high-income country, the majority of cases were advanced, with 45.5% presenting with Stage 4 disease.^28^ Most of our Stage 4 patients were offered palliation as can be expected in such advanced tumours.^29^

All patients with T1, T2 and T3 tumours were offered surgery primarily which concur with the findings of Smit et al.^3^ Sleeve resection was mainly done for T1 tumours. The T2 patient, who had a sleeve resection, ultimately needed a LTBR which in retrospect, should have been done primarily. Despite the fact that T1/T2 tumours may be resected by either sleeve resection or LTBR,^26^ one can argue that sleeve resection should be reserved for T1 tumours where the tumour is limited to the cartilaginous EAC. When the tumour extends onto the osseous bony canal, sleeve resection provides inadequate tumour resection and increases the risk of recurrence. The reason is that the skin of the bony ear canal is only 0.1–0.2 mm thick, and obtaining a clear margin is impossible without removing the bone as well.^8^ On the contrary, LTBR can achieve 100% disease clearance for T1 and T2 tumours and is therefore the treatment of choice.^3^ Most patients in our study had a lateral temporal bone resection as primary surgery, mainly for T2 and T3 tumours. However, we note the difference in failing to achieve clear margins for T3 tumours (80%) compared to T1/T2 tumours (20%). But as Saijo et al. noted, LTBR can be used for limited cases of T3 tumours which spare ‘the middle cranial fossa, eustachian tube, carotid canal, and petrous apex’, as it can be technically difficult to achieve clear surgical margins.^26^ The T4 patient who had the subtotal petrosectomy had primary SCC in the mastoid cavity. It was difficult to get clear margins as the extent of the primary could not be determined adequately, and concerns of further tumour seeding were present. He had involved margins post-operatively, and despite receiving chemoradiotherapy, went on to receive best supportive care. It is very difficult to achieve en bloc dissection of T4 tumours,^26^ and this is proof that T4 tumours should only be operated on if clear margins can be achieved.

In our study, parotid involvement was only found in three T3 cases. Half of the patients had a superficial parotidectomy, and this was independent of the tumour stage, unlike the findings of Magliocca et al., who found in their literature review that parotid involvement and parotidectomy increased in frequency with advancing tumour stage.^30^ There is no doubt that parotidectomy is indicated to achieve clear margins when there is direct or lymphatic tumour spread to the parotid gland. In our study, which comprised primary temporal bone tumours of varying histological types, 15% of cases had direct parotid gland invasion with tumour and 5% had intra-parotid nodal involvement. Current literature shows no difference in direct parotid involvement between cutaneous SCCs involving the temporal bone (9.7%)^31^ and primary temporal bone SCCs (8.2%).^3^ However, cutaneous SCCs have a higher rate of intra-parotid nodes (24.4%)^31^ than primary temporal bone SCCs (6.1%).^3^ The role of elective parotidectomy is controversial, with some authors suggesting superficial parotidectomy in cases of EAC SCC and others supporting total parotidectomy for all T3 and T4 tumours.^8^

All neck dissections were clinically and pathologically N0, which gave us no positive necks to compare with. Most neck dissections were performed in SCC patients, with levels 2 and 3 being the most dissected neck levels. Most authors advocate dissecting levels 2 and 3 as these are the most commonly affected neck levels and they facilitate vessel exposure for a microvascular free flap.^8^ Borsetto et al. found a 14% incidence of occult cervical metastases in temporal bone SCCs: 0%, 7%, 21% and 18% for T1, T2, T3 and T4 tumours, respectively, mostly to level 2, and they suggest levels 2 and 3 neck dissections in T3 and T4 temporal bone SCC patients only.^18^ Nonetheless, some authors recommend completing the dissection of levels I through V based on older studies that estimated the risk of occult metastases in these levels to be between 17% and 25%.^12^

The reconstruction of most of our surgical defects was performed using a temporalis muscle flap. This local flap suffices for most LTBR defects where the tumour does not extend beyond the pinna. However, for larger defects, a microvascular free flap may be necessary for bulk and skin coverage, or alternatively, where no microvascular expertise is available, a cervicofacial flap may be considered.^8^ We could not evaluate facial nerve repair since all our patients with facial nerve involvement had advanced disease and none were operable.

It is proven that positive resection margins is a negative prognostic factor.^28^ In our study, T3 tumours were more likely to have positive margins post-LTBR compared to T1 and T2 tumours. In a retrospective study by Piras et al., positive surgical margins were noted in 0%, 6.1%, 24.2% and 69.7% of Stages 1, 2, 3, and 4 tumours, respectively.^28^ This highlights the technical difficulty in achieving tumour-free margins in more advanced tumours. It also provides a rationale for post-operative radiotherapy in T3 tumours, especially when survival rates post-adjuvant radiotherapy have been found to be higher compared to surgery alone.^8^ Pre-operative chemoradiation therapy (CRT) has been shown to improve overall survival rates, in terms of improving local control rate and obtaining tumour-free margins, for T3 and T4 tumours. However, post-operative CRT has not been shown to improve overall survival.^8^ In our study, chemotherapy was used either as holding, palliative, or adjuvant CRT treatment, but never as definitive CRT. The latter has been shown to have equal or better survival rates than surgery with adjuvant radiotherapy for T3 and T4 temporal bone tumours.^8^ In a retrospective review by Ooka et al., concurrent chemoradiotherapy with docetaxel, cisplatin and 5-fluorouracil has been proposed as the first choice for unresectable advanced T4 carcinoma.^32^ Perhaps, this is an option that we should study at our institution.

We noted that both the mean overall survival and the disease-specific survival for our cohort were markedly lower than previously published outcomes.^32^ This can be attributed to the fact that most of our patients presented in advanced cancer stages. Moody et al. reported 2-year overall survival rates of 100%, 80%, 50% and 7% for T1, T2, T3 and T4 tumours, respectively,^8^ compared to our findings of 66.7%, 25%, 60% and 0%, respectively. The explanation for the surprisingly low survival rates for T2 tumours lies in the fact that all of our T2 patients suffered from multiple comorbidities and likely died from complications thereof. Saijo et al. reported 3-year disease-specific survival rates of 100%, 87.5% and 11.1% for T1-T2, T3 and T4 tumours, respectively,^23^ quite similar to our 2-year disease-specific survival rates findings for T1-T2 (100%) and T4 (0%) tumours, except for T3 tumours where our survival rate was much lower (50%), probably due to an 80% disease recurrence rate. Lassig et al. reported 2-year disease-free survival rates of 100%, 67% and 56% for T1–T2, T3 and T4 tumours, respectively,^33^ which is higher than our survival rates of 66.7%, 20% and 0%, respectively. This is possibly because Lassig et al. included both primary EAC tumours and tumours originating from adjacent sites, which could have resulted in better survival rates. Kaplan–Meier survival and log-rank analyses performed for sex and stage of disease did not show statistical significance, similar to the findings of Seligman et al.^23^ However, the latter did not study disease-specific and disease-free survival.

A high proportion (46.2%) of surgical resection margins were positive, which contributed to disease recurrence and poorer outcome. We observed higher rates of involved margins than in the literature (20% to 40.1%).^3,5,14,16,27^ We should be aggressive in surgically resecting temporal bone tumours, and at the same time, achieve an acceptable level of morbidity. There is no doubt that disease recurrence is a negative predictor of survival outcomes.^14^ Our findings concur with those of Hintze et al., where disease recurrence occurred in 26.8% of their patients, and mean time to recurrence was 9.7 months.^32^ One can therefore hypothesise that temporal bone tumours should be treated aggressively to minimise recurrence.^25^

### Limitations

While the objective of our study was to provide an overview of all patients presenting with temporal bone cancers, the main limitations were the small sample size and the retrospective single-institution nature of the study, with reliance on existing patient records. The small sample size was unlikely to be sufficiently powered to influence outcome analysis. Unfortunately, we did not evaluate the severity of patient comorbidities which might have influenced survival analysis, due to missing information from patient records. We also included a mixed group of temporal bone cancer histological diagnoses, with each tumour subtype differing in its pathologic behaviour, aggressiveness and spread. Hence, survival outcomes and prognostic factors should be interpreted with caution. In addition, larger, long-term, multicentre prospective studies are needed to develop a prognostic staging system and an evidence-based treatment protocol. This will increase our understanding of the disease and will improve survival outcomes.

## Conclusion

Temporal bone carcinomas display aggressive behaviour and are challenging to treat. With regard to surgical procedures aimed at achieving clear margins, sleeve resection should be reserved for T1 tumours where the tumour is limited to the cartilaginous EAC; parotidectomy should be done when there is direct or lymphatic tumour spread to the parotid gland or where the anterior ear canal wall is involved. For T2, T3 and T4 SCC without nodal metastasis, neck dissection of levels 2 and 3 should be done. Adjuvant radiotherapy should be given for at least T3 SCCs and/or where resection margins are positive.
